# Vedolizumab Attenuates Immune-Checkpoint-Therapy-Induced Infliximab-Refractory Colitis

**DOI:** 10.3390/diagnostics12020480

**Published:** 2022-02-13

**Authors:** Ayaka Kaneoka, Etsuko Okada, Hitomi Sugino, Natsuko Saito-Sasaki, Daisuke Omoto, Motonobu Nakamura

**Affiliations:** Department of Dermatology, University of Occupational and Environmental Hearth, 1-1 Iseigaoka, Yahatanishi-ku, Kitakyusyu 807-8555, Japan; e-okada@med.uoeh-u.ac.jp (E.O.); hitomi-sugino@med.uoeh-u.ac.jp (H.S.); natsuko-saito@med.uoeh-u.ac.jp (N.S.-S.); daisuke-omoto@med.uoeh-u.ac.jp (D.O.); motonaka@med.uoeh-u.ac.jp (M.N.)

**Keywords:** malignant melanoma, immune checkpoint inhibitor, immune-related adverse events, colitis, infliximab-refractory, vedolizumab

## Abstract

Immune checkpoint inhibitors (ICIs), such as nivolumab and ipilimumab, have drastically changed treatments of advanced melanoma. However, ICI-related enterocolitis is often the most common adverse event, and represents the main reason for ICI discontinuation and mortalities. Here, we report the case of a metastatic melanoma treated with vedolizumab for ICI-induced colitis. A 67-year-old man treated with ipilimumab and nivolumab developed ICI-induced colitis and grade 3 diarrhea refractory to methylprednisolone and infliximab. After his third dose of vedolizumab, oral prednisolone ceased, and the colitis had completely resolved with no recurrence. This case report supports vedolizumab use in treating severe colitis which failed to resolve with first- and second-line immunosuppressive therapy.

## 1. Introduction

Combined blockade of cytotoxic T-lymphocyte antigen-4 (CTLA-4) and programmed cell death protein-1 (PD-1) is the standard treatment in patients with metastatic melanoma, and it improves response rates and overall survival compared with PD-1 inhibitors alone [1]. The currently achieved response to the ICI treatment of melanoma patients reached 58% for a combination of ipilimumab and nivolumab, and the 5-year survival rate was reported to be 52% [2,3,4]. However, the use of combined immune-checkpoint inhibitors (ICIs), ipilimumab and nivolumab, also lead to a higher rate of toxicity, including gastrointestinal immune-related adverse events (irAE), which tend to occur earlier when compared to monotherapy [1,5]. The irAE-associated mortality is 1.23% of patients who received immunotherapy, 37% of which were caused by colitis [6]. Grade 3/4 colitis occurs in 17% of patients on combined immunotherapy compared to 4% on nivolumab monotherapy [1]. Recently, ESMO clinical practice guidelines reported the therapy of ICI-induced colitis. For grade 1 diarrhea, symptomatic treatment with loperamide and rehydration is advised. For grade 2 diarrhea, steroid therapy with either budesonide or 1 mg/kg oral prednisone is recommended. In grade3/4 diarrhea, high-dose intravenous steroids should be administered. If no improvement is seen after three to five days, a dose of infliximab can be used [5]. In intractable cases with these treatments, vedolizumab is currently used. Vedolizumab is a humanized murine antibody with activity against the α4β7-integrin heterodimer on the surface of CD4+ T cells [7]. The α4β7-integrin binds to its ligand MAdCAM-1, which is expressed on the endothelial surface of venules within the gut and its associated lymphoid tissue, facilitating the trafficking of T cells into the gut mucosa. By blocking the interaction between α4β7-integrin and MadCAM-1, vedolizumab prevents T cells from binding and homing into the inflamed bowel mucosa, which can explain its efficacy in ICI-mediated enterocolitis [7]. Vedolizumab is currently approved for IBD treatment but is off-label for ICI-induced colitis.

## 2. Case Report

**Figure 1:**
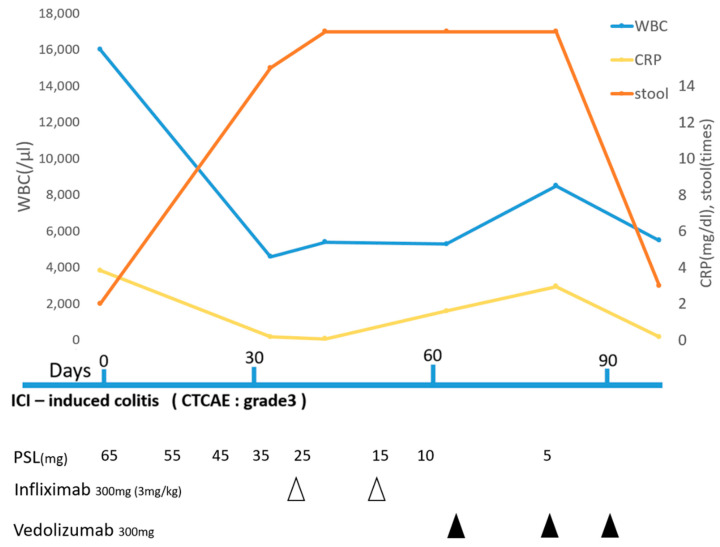
Clinical course in patient. Refractory to corticosteroid in times of stools, increased stability of CRP at infliximab treatment and, a reduction in CRP levels and diarrhea after vedolizumab treatment were observed.

**Figure 2:**
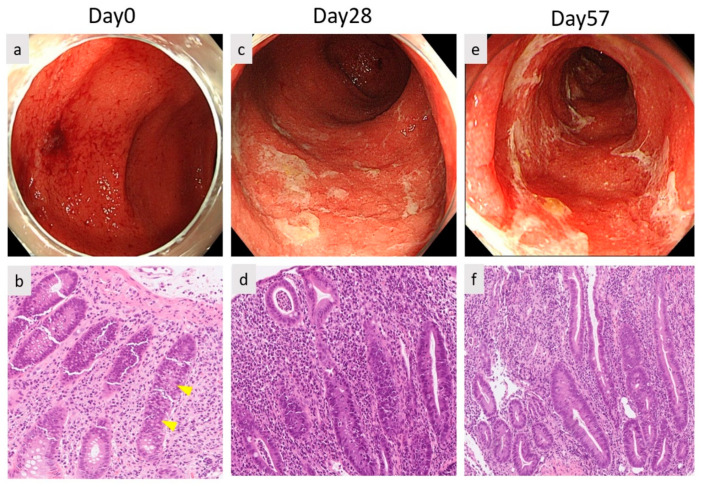

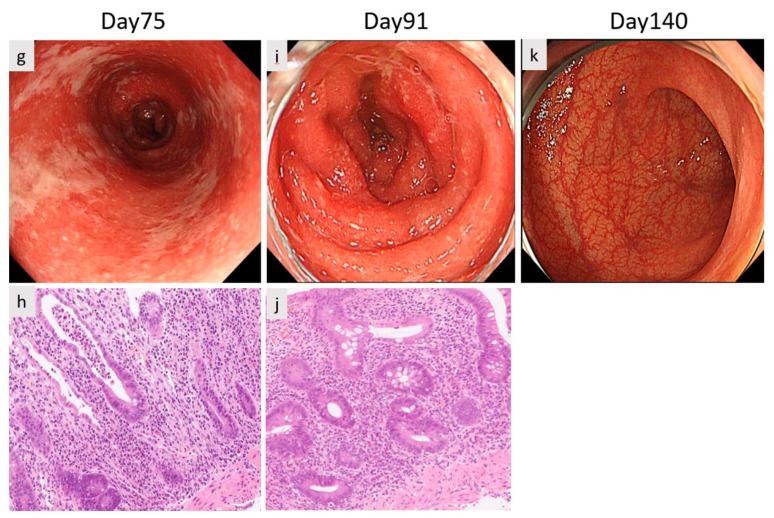
Endoscopic images of colon and histological findings. (**a**) Day 0; diffuse inflammation in the descending colon with erosions, erythema, friability and a loss of vascularity. (**b**) A gut biopsy showed the presence of inflammatory cells (neutrophils, plasma cells and histiocyte) in the lamina propria and crypt abscesses. (**c**,**d**) Day 28. (**e**,**f**) Day 57 (after the second dose of infliximab). (**g**,**h**) Day 75 (after the first dose of vedolizumab). (**i**,**j**) Day 91 (after the third dose of vedolizumab). (**k**) Day 140.

The subject was a 67-year-old male, diagnosed with malignant melanoma of the left heel. After the tumor resection, Breslow’s thickness of tumor was 5.0 mm, and one metastatic left inguinal lymph node was identified. The clinical staging was stage Ⅲc (pT4bN2cMo), and the tumor was negative for PD-L1 and wild type *BRAF V600E/K.* With high disease burden and good performance status, he was administered adjuvant immunotherapy with nivolumab every two weeks. After cycle 16, progressive metastases were observed in the external iliac lymph nodes and lungs. Therefore, he received combined immunotherapy with ipilimumab and nivolumab. However, this was discontinued 20 days after the first cycle due to ICI-induced grade 1 interstitial pneumonia. Moreover, 23 days after, he developed grade 3 severe non-bloody diarrhea according to CTCAE. Laboratory examination showed an elevated C-reactive protein level (CRP) (5.24 mg/dL) and white blood cell count (WBC) (14,200/μL) (Figure 1). No pathogenic bacterium grew in a stool culture, so we excluded infection. Computed tomography (CT) showed intestinal wall thickening and surrounding adipose tissue turbidity. Colonoscopy revealed diffuse moderate inflammation in the descending colon characterized by erosions, erythema, friability, and a loss of vascularity (Figure 2a). Biopsy from his intestine showed that the neutrophils, plasma cells and histiocyte infiltrated the lamina propria and made crypt abscesses (Figure 2b). Thus, he was diagnosed with ICI-induced colitis. An amount of 1 mg/kg/day (65 mg/day) of oral prednisolone was administered and weaned by 10 mg/day each week. Laboratory examination showed a decreased CRP (0.18 mg/dL) and WBC (4600/μL). Attempts of steroid weaning failed, with recurrent problematic diarrhea each time the dose went below 35 mg/day. Furthermore, 28 days after the onset, the first dose of infliximab was administered, followed by a second dose at week two. However, no improvement was observed (Figure 2c,d). Then, 58 days after colitis onset (Figure 2e,f), we decided to treat the patient with a first dose of 300 mg vedolizumab (Figure 2g,h). After the second dose at week two, he had only three formed bowel movements per day with a progressive reduction in CRP (Figure 1), so predonisolone was tapered down to 5 mg/day. Colonoscopy showed mild mucosal inflammation with no ulceration. After the third dose at week 6 (Figure 2i,j), no further maintenance vedolizumab was administered thereafter (Figure 2k). ICI was discontinued, so he was treated with dacarbazine.

## 3. Discussion

Combined immunotherapy with ipilimumab and nivolumab is an established approach in higher risk metastatic melanoma. Gastrointestinal toxicity is the most prevalent irAE seen in immunotherapy [1]. The onset of gastrointestinal symptoms may occur at any time during the 1–10 infusions of anti-CTLA4 [8]. Enterocolitis may even occur several months after the last dose of ipilimumab [9]. The half-life of ipilimumab is two weeks. However, the biological effect may persist long after drug clearance. In this case, the tumor showed no response to treatment with nivolumab alone, the patient received combined immunotherapy. However, 23 days after the treatment, he developed grade 3 severe non-bloody diarrhea.

The risk of colitis depends mainly on three aspects: the ICI, the tumor, and the patient. When taking underlying cancer into consideration, patients with melanoma seem to have a higher risk of developing colitis than those who receive ICIs for other types of cancer [10]. Up to 41% of patients with melanoma who received ipilimumab develop all-grade diarrhea compared with 25–27% of patients with lung cancer. Similarly, the incidence of diarrhea in patients with lung cancer receiving PD-1/PDL1 agents ranges from 5% to 14% compared with 10–22% in patients with melanoma [10]. Patient characteristics play a crucial role in determining the risk of developing colitis. Studies have shown that the gut microbiome influences both the efficacy of PD-1 based immunotherapy and the risk of developing colitis [11,12,13,14,15]. This finding leads to the advent of fecal microbiota transplantation as an effective treatment of refractory colitis [16].

The most common symptom of anti-CTLA4-induced enterocolitis is diarrhea [17,18,19,20], and 92% of the patients had diarrhea [21]. Other presenting symptoms are abdominal pain, hematochezia, weight loss, fever, and vomiting [21]. These symptoms are often the first event leading to the discontinuation of therapy. In managing colitis, we must exclude infection with stool culture and bloody cytomegalovirus antigen, as well as contemplating the potential for gastrointestinal metastases. CT imaging, colonoscopy, and biopsy from his intestine supported the evidence of ICI-induced colitis. ESMO clinical practice guidelines reported the therapy of ICI-induced colitis. The first approach to grade 3/4 diarrhea colitis is treatment of intravenous methylprednisolone 1–2 mg/kg and obtaining a gastroenterology consult and colonoscopy [5]. Up to half of these cases will be steroid-refractory, necessitating further immunomodulation [22]. If no improvement is shown within 72 h, 5 mg/kg infliximab should be considered [5]. In the difficult cases with these treatments, vedolizumab becomes a third option for ICI-induced colitis. Vedolizumab’s landmark trial was in patients with ulcerative colitis and Crohn’s disease refractory to standard treatments, where it was administered intravenously at zero, two, and six weeks, then every eight weeks as maintenance [23,24]. It had a tolerable side effect profile with mild nasopharyngitis, headache, arthralgia, nausea and fatigue, but no increased rates of infection [23,24,25]. Vedolizumab is an attractive option due to its gut selectively. Vedolizumab is less likely to mitigate the antitumor effect of ipilimumab and to have the additional benefit of not heightening the risk of opportunistic infection or secondary malignancies in an already vulnerable patient as is possible with infliximab use [26].

In the context of ICI-induced infliximab-refractory colitis, we found some published case reports assessing the use of vedolizumab [27,28,29,30]. Regardless of staging and cycles of ICI, two or three doses of vedolizumab improved colitis without any side effects in these cases. In our case, two doses of infliximab led to no improvement, so we decided to administer vedolizumab. After the third vedolizumab treatment, his colitis was gradually improved without any additional treatment. Other studies have shown that vedolizumab is associated with a clinical response rate of 95% in patients with ICI-induced colitis who did not receive prior infliximab, with an endoscopic remission rate > 50% [30]. The vedolizumab-alone group had the lowest rate of colitis recurrence compared with the infliximab-alone group, and patients received combined selective immunosuppressive therapies sequentially [31]. It might be better to switch to vedolizumab treatment in recurrent refractory cases against infliximab.

One study showed that the incidence of recurrent colitis is lower in patients who resumed an anti-PD-1/L1 agent than in those who resumed an anti-CTLA-4 agent [32]. Considering the higher risk of recurrence associated with CTLA-4 inhibitors, we recommend considering permanently discontinuing CTLA-4 inhibitors [33]. Patients who required immunosuppressive therapy for initial colitis were more likely to experience colitis recurrence after the resumption of ICI therapy [32]. Likewise, a long duration of colitis symptoms in the initial episode was associated with a higher risk of colitis recurrence [32]. Patients who develop irAEs tend to have high rates of response to ICI therapy, which are sustained and may obviate ICI resumption [34,35,36]. In real life settings, treatment decisions are made on a per-patient basis, depending on comorbid conditions and cancer therapy options. We switched to dacarbazine for his treatment, as nivolumab was ineffective and ipilimumab was at the risk of relapse of colitis.

## 4. Conclusions

This case report supports vedolizumab use in severe immune-related colitis refractory to standard immunosuppression. There is a growing need for developing evidence-based and increasingly optimized irAE management algorithms, taking long-term cancer-treatment strategy and overall survival into account.

## Data Availability

The original contributions presented in the study are included in the article/supplementary material. Further inquiries can be directed to ak---1007@med.uoeh-u.ac.jp.
